# “Real World” Eligibility for Sacubitril/Valsartan in Unselected Heart Failure Patients: Data from the Swedish Heart Failure Registry

**DOI:** 10.1007/s10557-019-06873-1

**Published:** 2019-03-23

**Authors:** Joanne Simpson, L. Benson, P. S. Jhund, U. Dahlström, J. J. V. McMurray, L. H. Lund

**Affiliations:** 1 British Heart Foundation Glasgow, Institute of Cardiovascular and Medical Sciences, University of Glasgow, University Avenue, Glasgow, G12 8TA UK; 2 Department of Clinical Science and Education, Södersjukhuset, Stockholm, Sweden; 3 Karolinska Institutet, Stockholm, Sweden; 4 Department of Cardiology and Department of Medicine and Health Sciences, Linköping University, Linköping, Sweden; 5 Department of Medicine, Karolinska Institutet and Heart and Vascular Theme, Karolinska University Hospital, Stockholm, Sweden

**Keywords:** Heart failure, Eligibility, Sacubitril-valsartan, Real-world population, PARADIGM-HF

## Abstract

**Purpose:**

PARADIGM-HF demonstrated the superiority of sacubitril/valsartan over enalapril in patients with heart failure and reduced ejection fraction (HF-REF). How widely applicable sacubitril/valsartan treatment is in unselected patients with HF-REF is not known. We examined eligibility of patients with HF-REF for treatment with sacubitril/valsartan, according to the criteria used in PARADIGM-HF, in the Swedish Heart Failure Registry (SwedeHF).

**Methods:**

Patients were considered potentially eligible if they were not hospitalized, had symptoms (NYHA class II–IV) and a reduced LVEF (≤ 40%), and were prescribed an angiotensin-converting enzyme inhibitor (ACEI) or angiotensin receptor blocker (ARB) at a dose equivalent to enalapril ≥ 10 mg daily. In these patients, we evaluated further eligibility according to the main additional PARADIGM-HF inclusion criteria.

**Results:**

Of 12,866 outpatients in NYHA functional class II–IV with an LVEF ≤ 40%, 9577 were prescribed at least 10 mg of enalapril (or equivalent) daily. Complete additional data were available for 3099 of these patients (32.4%) and of them 75.5% were potentially eligible for treatment with sacubitril/valsartan. The most common reason for ineligibility was a low natriuretic peptide level (*n* = 462, 14.9%). Only a small proportion of patients were ineligible due to low eGFR or serum potassium level. Because only 78% of patients were taking ≥ 10 mg enalapril or equivalent daily, only 58.9% of all patients (75.5% of 78%) were eligible for sacubitril/valsartan.

**Conclusions:**

Between 34 and 76% of symptomatic patients with HF-REF in a ‘real world’ population are eligible for treatment with sacubitril/valsartan, depending on background ACEI/ARB dose. The most common reason for ineligibility is a low natriuretic peptide level.

**Electronic supplementary material:**

The online version of this article (10.1007/s10557-019-06873-1) contains supplementary material, which is available to authorized users.

## Introduction

The angiotensin receptor neprilysin inhibitor (ARNI) sacubitril/valsartan combines an angiotensin receptor blocker (ARB) with a neprilysin inhibitor. The Prospective comparison of ARNI with ACEI to Determine Impact on Global Mortality and morbidity in Heart Failure trial (PARADIGM-HF) demonstrated the superiority of the sacubitril/valsartan (formerly known as LCZ696) over the angiotensin-converting enzyme inhibitor (ACEI) enalapril in patients with heart failure and reduced ejection fraction (HF-REF) [1]. In PARADIGM-HF, sacubitril/valsartan reduced the risk of the primary composite outcome of cardiovascular death or heart failure hospitalization by 20%, each of the components of that endpoint by a similar amount and all-cause death by 16% [1]. As a result, international guidelines now recommend the use of sacubitril/valsartan as a preferred alternative to an ACEI or ARB [2, 3]. How widely applicable sacubitril/valsartan treatment is in unselected patients with HF-REF is uncertain. We used the Swedish Heart Failure Registry (SwedeHF) to examine eligibility of patients for sacubitril/valsartan in an unselected nationwide cohort. We identified individuals fulfilling the main inclusion criteria for the PARADIGM-HF trial, i.e., ambulatory patients with persisting symptoms (NYHA class II–IV), low ejection fraction (EF) (< 40%), and elevated natriuretic peptides, despite treatment with an ACEI or an ARB (scenario 1). To be enrolled in the PARADIGM-HF active run-in phase, patients had to be treated with a dose of ACE inhibitor or ARB equivalent to enalapril 10 mg daily—we further identified the subset of patients in SwedeHF treated in this way (scenario 2). Finally, as patients had to tolerate up-titration to a daily dose of 20 mg enalapril during the active run in, we also identified the proportion of patients in SwedeHF taking a dose of ACE inhibitor or ARB equivalent to this dose of enalapril (scenario 3).

## Methods

### Patients

Patients with heart failure included in SwedeHF between July 2005 and December 2012 were studied. This nationwide internet-based registry has been described in detail [4]. Patients are included in the registry based upon a clinical diagnosis of heart failure and registered at discharge from hospital or following an outpatient visit. Data on over 100 primary and derived variables are collected, including clinical findings, laboratory measurements, and medications. Left ventricular EF is reported in the registry as less than 30%, 30–39%, 40–49%, and 50% or greater. For this study, we included only patients with an EF < 40%, and only the most recent patient encounter was considered.

### Eligibility for Sacubitril/Valsartan

The entry criteria for PARADIGM-HF were used to determine eligibility status for each patient [5]. Patients were considered potentially eligible and included in the denominator in this study if they were not hospitalized (because only ambulatory patients were included in PARADIGM-HF; potentially eligible patients in both an outpatient and inpatient setting are shown in Supplementary Table 1) and had symptoms (NYHA functional class II–IV) and a low EF (< 40%) and were prescribed an ACEI or an ARB at a dose equivalent to enalapril 10 mg daily. The dose equivalents for these analyses were defined PARADIGM-HF inclusion criteria [5].

In these patients, we evaluated further eligibility in relation to the main additional PARADIGM-HF inclusion criteria, i.e., plasma B-type natriuretic peptide (BNP) ≥ 150 pg/ml (or N-terminal pro-BNP [NT-proBNP] ≥ 600 pg/ml) or, in patients hospitalized for HF in the preceding 12 months, BNP ≥ 100 pg/ml or NT-proBNP ≥ 400 pg/ml; and exclusion criteria, i.e., systolic blood pressure (SBP) < 100 mmHg, serum potassium concentration > 5.2 mmol/l, or an estimated glomerular filtration rate (eGFR) < 30 ml/min/1.73 m^2^. For the main analyses, we only included patients with complete data entries for these inclusion and exclusion criteria. In a consistency analysis, missing data were imputed by multivariate imputation using chained equations. The extent of missing data is shown in Table 2 of the Supplementary Appendix.

### Background ACE Inhibitor Dose

In PARADIGM-HF, patients had to be treated with an ACE inhibitor or an ARB at a dose equivalent to enalapril 10 mg/day for at least 4 weeks before the screening visit and then entered an active run-in phase where they received open-label enalapril which was titrated to a dose of 10 mg bid over a period of 3 to 5 weeks. Patients tolerating enalapril 10 mg bid were then started on sacubitril/valsartan 49/51 mg bid, which, if tolerated, was increased to a dose of 97/103 mg bid after 1–2 weeks. If tolerated, sacubitril/valsartan 97/103 mg bid was continued for a further 2–4 weeks, at which point patients were randomized to enalapril 10 mg bid or sacubitril/valsartan 97/103 mg bid. At each step, during the two run-in periods, patients had to demonstrate satisfactory renal function and serum potassium concentration, as well as a systolic blood pressure before randomization of at least 95 mmHg and no symptoms of hypotension. For this reason, we did separate analyses of otherwise eligible patients depending on whether they were on a background ACE inhibitor/ARB dose equivalent to 10 mg daily and 20 mg daily.

### Ethical Considerations

Establishment of SwedeHF, and the present analysis, was approved by a multisite ethics committee. Patients in Sweden are informed of the intent to include their data in the national registry and may choose to opt out of this.

### Statistical Analysis

For baseline characteristics, categorical data are presented as numbers and percentages and parametric data as mean and standard deviation (medians and interquartile ranges are used for data that are not normally distributed).

Continuous variables were modeled with linear regression and dichotomous variables were imputed via logistic regression. In sensitivity analyses to avoid bias from data not missing at random, multiple imputation was used to handle missing data.

We considered a two-sided *p* value < 0.05 as significant. All analyses were conducted using Stata version 14.1 (StataCorp LP, College Station, Texas).

## Results

### Baseline Characteristics

We studied 51,060 patients included in SwedeHF. The most recent patient encounter was as an outpatient in 22,822 patients (potentially eligible patients in both an outpatient and inpatient setting are shown in Supplementary Table 1). Of these, 74 were excluded because of death on the day of their visit, a further 8512 because of an EF ≥ 40%, and among the remainder, 1370 were excluded because they were NYHA functional class I. This left 12,866 patients in NYHA functional class II–IV with an EF < 40%, i.e., potentially eligible for treatment with sacubitril/valsartan; their baseline characteristics are shown in Table 1.

**Table 1:** Baseline characteristics of patients in the Swedish Heart Failure Registry potentially eligible for sacubitril/valsartan (outpatients, NYHA II-IV and EF < 40%)

	Whole cohort	Patients with complete eligibility data	Patients with incomplete eligibility data
	*n* = 12,866	*n* = 3963	*n* = 8903
Age (years)	72.0 ± 11.8	69.7 ± 11.9	73.0 ± 11.7
Female	3906 (30.4%)	1041 (26.3%)	2865 (32.2%)
Duration of HF ≥ 6 months	7487 (58.4%)	2180 (55.1%)	5307 (59.8%)
Systolic BP (mmHg)	125.1 ± 20.6	122.6 ± 19.72	126.3 ± 20.9
Diastolic BP (mmHg)	72.7 ± 11.6	72.2 ± 11.62	73.0 ± 11.5
Heart rate (bpm)	71.2 ± 14.2	71.0 ± 13.93	71.3 ± 14.3
BMI (kg/m^2^)	27.2 ± 5.3	27.3 ± 5.3	27.1 ± 5.3
NYHA functional class			
II	6201 (56.3%)	2210 (55.8%)	3991 (56.6%)
III	4573 (41.5%)	1672 (42.2%)	2901 (41.1%)
IV	242 (2.2%)	81 (2.0%)	161 (2.3%)
Medical history			
Hypertension	5857 (47.1%)	1723 (44.5%)	4134 (48.2%)
Type 1 diabetes mellitus	99 (0.8%)	47 (1.2%)	52 (0.6%)
Type 2 diabetes mellitus	2935 (22.8%)	892 (22.5%)	2043 (22.9%)
Atrial fibrillation/flutter	5695 (44.6%)	1722 (43.6%)	3973 (45.0%)
Ischemic heart disease	6034 (49.4%)	1758 (47.0%)	4276 (50.5%)
Valvular heart disease	2074 (16.8%)	654 (16.7%)	1420 (16.9%)
Left bundle branch block	2449 (23.0%)	862 (26.6%)	1587 (21.4%)
Treatments			
ACE inhibitor	8586 (66.9%)	2765 (69.8%)	5821 (65.6%)
ARB	3578 (27.9%)	1172 (29.6%)	2406 (27.1%)
ACE inhibitor or ARB	11,719 (91.1%)	3800 (95.9%)	7919 (88.9%)
Beta-blocker	11,391 (88.7%)	3736 (94.3%)	7655 (86.3%)
Diuretic	9953 (77.6%)	3041 (76.9%)	6912 (78.0%)
Digoxin	2130 (16.6%)	644 (16.3%)	1486 (16.8%)
MRA	4323 (33.8%)	1540 (39.0%)	2783 (31.4%)
Pacemaker	971 (7.6%)	282 (7.1%)	689 (7.9%)
CRT	657 (5.1%)	308 (7.8%)	349 (3.9%)
ICD	373 (2.9%)	151 (3.8%)	222 (2.5%)
Laboratory values			
Hemoglobin (g/dl)	135.3 ± 16.2	136.8 ± 15.9	134.7 ± 16.2
Creatinine (μmol/l)	106.22 ± 47.5	103.90 ± 43.1	107.26 ± 49.3
Potassium (mmol/l)	4.24 ± 0.43	4.27 ± 0.42	4.23 ± 0.44
NT-proBNP (pg/ml)	2120 [933–4607]	2159 [990–4655]	2035 [813–4447]
BNP (pg/ml)	450 [154–1100]	507 [176–1186]	380 [133–894]

*ACE* angiotensin-converting enzyme, *ARB* angiotensin receptor blocker, *BP* blood pressure, *BMI* body mass index, *BNP* brain natriuretic peptide, *CRT* cardiac resynchronization therapy, *HF* heart failure, *ICD* implantable cardioverter defibrillator, *MRA* mineralocorticoid receptor antagonist, *NYHA* New York Heart Association

Of these 12,866 patients, 3963 (31%) had complete data with respect to the PARADIGM-HF inclusion/exclusion criteria examined. Data were missing for BNP in 12,007 patients, NT-proBNP in 7838 (both natriuretic peptides were missing in 6998 patients), potassium in 3629, creatinine in 1687, and systolic blood pressure in 251 patients.

### Scenario 1: Eligibility of Patients Regardless of Background ACE Inhibitor or ARB Dose

**Fig. 1:**
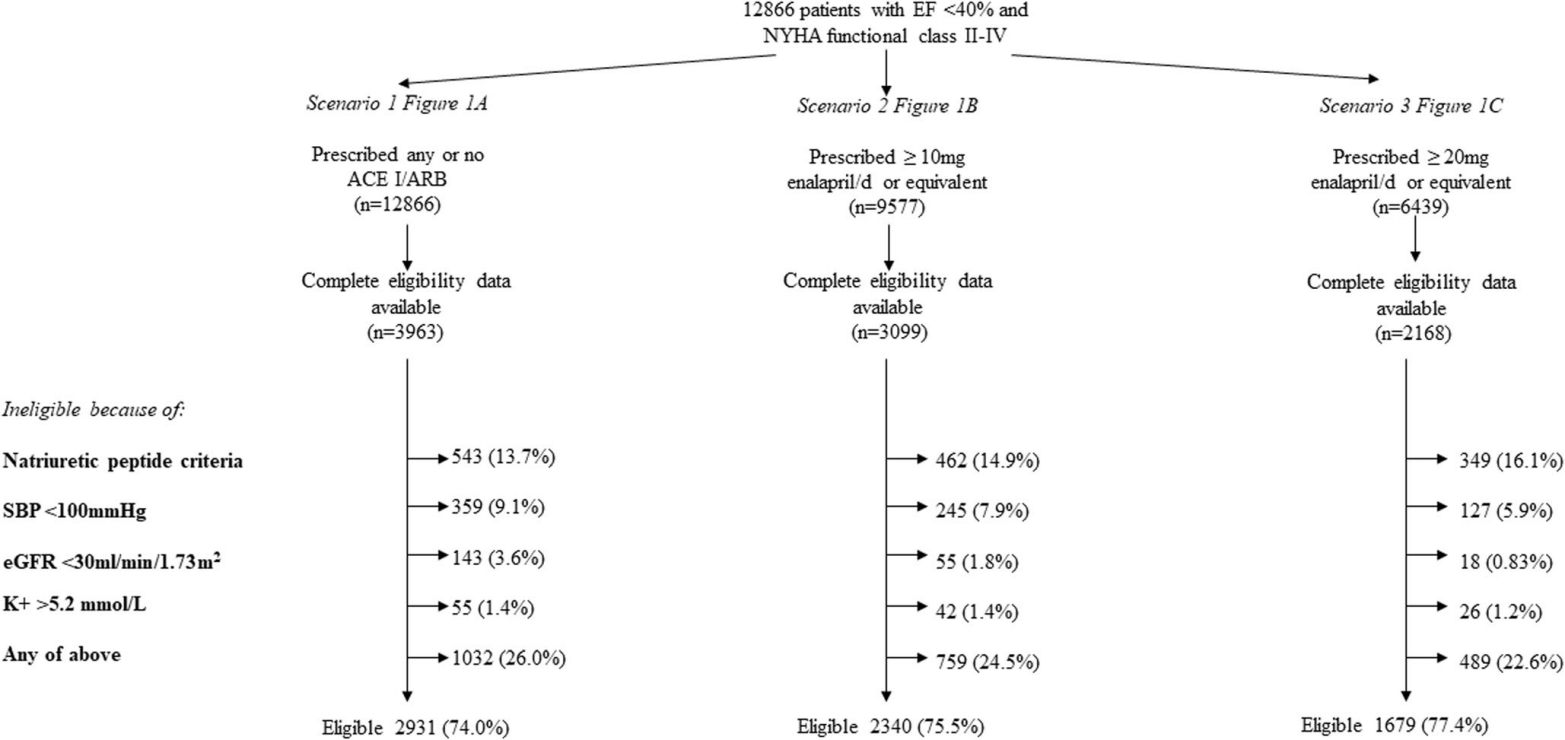
Different eligibility scenarios tested

As stated above, among the 12,866 patients with symptomatic HF-REF taking no or any dose of ACEI/ARB and potentially eligible for treatment with sacubitril/valsartan, complete data based on the PARADIGM-HF inclusion and exclusion criteria were available for 3963 (31%) patients. Of these 3963 patients, 1032 (26%) were ineligible for treatment with sacubitril/valsartan, i.e., 74% were eligible for treatment (Fig. 1a). The most common reason for ineligibility was a low natriuretic peptide level (*n* = 543, 13.7% of 3963 eligible patients with complete data), followed by a low systolic blood pressure (*n* = 359, 9.1%). Only a small proportion of patients were ineligible due to a low eGFR (*n* = 143, 3.6%) or high serum potassium level (*n* = 55, 1.4%).

A sensitivity analysis imputing missing values (increasing the number of patients eligible for analysis to 8903) estimated that 26% of patients were ineligible for treatment, i.e., 74% were eligible for treatment.

### Scenario 2: Eligibility of Patients Prescribed at Least 10 Mg Enalapril Daily, or Equivalent

Among the 9577 (74.4%) patients with symptomatic systolic heart failure taking at least 10 mg of enalapril (or equivalent) daily, and potentially eligible for treatment with sacubitril/valsartan, complete data based on the PARADIGM-HF inclusion and exclusion criteria were available for 3099 (32.4%) patients. Of these 3099 patients, 759 (24.5%) were ineligible for treatment with sacubitril/valsartan, i.e., 75.5% were potentially eligible for treatment (Fig. 1b). The most common reason for ineligibility was a low natriuretic peptide level (*n* = 462, 14.9% of 3099 eligible patients with complete data), followed by a low systolic blood pressure (*n* = 245, 7.9%). Only a small proportion of patients were ineligible due to a low eGFR (*n* = 55, 1.8%) or a high serum potassium level (*n* = 42, 1.4%).

A sensitivity analysis imputing missing values (*n* = 6478) estimated that 24.54% of patients were ineligible for treatment, i.e., 75.5% were potentially eligible for treatment with sacubitril/valsartan.

Because 3099 of 3963 (78.2%) of patients were taking ≥ 10 mg enalapril or equivalent daily, only 59.0% of all patients (75.5% of 78.2%) were eligible for sacubitril/valsartan. The equivalent figure using the imputed dataset was 52.44% (75.5% of 72.8%).

### Scenario 3: Eligibility of Patients Prescribed at Least 20 Mg Enalapril Daily, or Equivalent

Among the 6439 patients with symptomatic systolic heart failure taking at least 20 mg of enalapril (or equivalent) daily, and potentially eligible for treatment with sacubitril/valsartan, complete data based on the PARADIGM-HF inclusion and exclusion criteria were available for 2168 (33.7%) patients. Of these 2168 patients, 489 (22.6%) were ineligible for treatment with sacubitril/valsartan, i.e., 77.4% were potentially eligible for treatment (Fig. 1c). The most common reason for ineligibility was a low natriuretic peptide level (*n* = 349, 16.1% of 2168 eligible patients with complete data) followed by a low systolic blood pressure (*n* = 127, 5.9%). Only a small proportion of patients were ineligible due to a low eGFR (*n* = 18, 0.8%) or a high serum potassium level (*n* = 26, 1.2%).

A sensitivity analysis imputing missing values (*n* = 4271) estimated that 22.6% of patients were ineligible for treatment, i.e., 77.4% were potentially eligible for treatment.

Because only 54.7% of patients were taking ≥ 20 mg enalapril or equivalent daily, only 42.3% of all patients (77.4% of 55%) were eligible for sacubitril/valsartan. The equivalent figure using the imputed dataset was 34.7% (77% of 48%).

### Differences Between Baseline Characteristic by Eligibility Status

In comparing eligible vs. non-eligible patients, we used scenario 2 (10 mg of enalapril or equivalent daily, Table 2). Patients eligible for sacubitril/valsartan were more likely to be older, have heart failure for shorter duration, higher blood pressure, faster heart rate, and lower BMI. These patients were also more likely to have a history of hypertension, valvular heart disease, and atrial fibrillation and to be treated with a diuretic or digoxin. Eligible patients had lower creatinine and potassium and higher natriuretic peptides.

**Table 2:** Baseline characteristics of patients taking enalapril 10 mg daily or equivalent in the Swedish Heart Failure Registry by eligibility status

	Patients eligible for sacubitril/valsartan	Patients not eligible for sacubitril/valsartan	*P* value
	*n* = 2340	*n* = 759	
Age (years)	69.8 ± 11.4	65.2 ± 12.4	*p* < 0.0001
Female	615 (26.3%)	205 (27.0%)	*p* = 0.69
Duration of HF ≥ 6 months	1193 (51.1%)	469 (61.9%)	*p* < 0.0001
Systolic BP (mmHg)	126.4 ± 17.7	114.9 ± 22.2	*p* < 0.0001
Diastolic BP (mmHg)	74.0 ± 11.2	69.0 ± 11.9	*p* < 0.0001
Heart rate (bpm)	70.9 ± 14.2	68.9 ± 12.5	*p* = 0.0006
BMI (kg/m^2^)	27.3 ± 5.2	28.1 ± 5.6	*p* = 0.0119
NYHA functional class			*p* = 0.10
II	1352 (57.8%)	446 (58.8%)	
III	957 (40.9%)	295 (38.9%)	
IV	31 (1.3%)	18 (2.4%)	
Medical history			
Hypertension	1094 (48.0%)	289 (38.7%)	*p* < 0.0001
Atrial fibrillation/flutter	1051 (50.0%)	263 (34.7%)	*p* < 0.0001
Ischemic heart disease	1025 (46.4%)	315 (44.1%)	*p* = 0.28
Valvular heart disease	394 (17.0%)	80 (10.6%)	*p* < 0.0001
Left bundle branch block	2449 (27.1%)	165 (26.0%)	*p* = 0.57
Treatment			
Beta-blocker	2225 (95.1%)	731 (96.3%)	*p* = 0.16
Diuretic	1811 (77.7%)	537 (70.8%)	*p* = 0.0001
Digoxin	416 (17.8%)	85 (11.2%)	*p* < 0.0001
MRA	955 (40.9%)	337 (44.6%)	*p* = 0.07
Pacemaker	155 (6.6%)	32 (4.2%)	*p* = 0.0155
CRT	172 (7.4%)	72 (9.5%)	*p* = 0.0576
ICD	86 (3.7%)	36 (4.7%)	*p* = 0.1886
Laboratory values			
Hemoglobin (g/dl)	137.5 ± 15.6	138.4 ± 15.8	*p* = 0.15
Creatinine (μmol/l)	97.0 ± 27.7	102.7 ± 44.2	*p* < 0.0001
Potassium (mmol/l)	4.25 ± 0.39	4.31 ± 0.47	*p* = 0.0008
NT-proBNP (pg/ml)	2290 [1270–4486]	451 [247–2909]	*p* < 0.0001
BNP (pg/ml)	625 [321–1357]	93.0 [53–248]	*p* < 0.0001

*BP* blood pressure, *BMI* body mass index, *BNP* brain natriuretic peptide, *CRT* cardiac resynchronization therapy, *HF* heart failure, *ICD* implantable cardioverter defibrillator, *MRA* mineralocorticoid receptor antagonist, *NYHA* New York Heart Association

## Discussion

Our results from one of the largest contemporary “real world” cohorts of patients with heart failure show that between 34 and 74% of outpatients with a reduced EF and persisting symptoms are eligible for treatment with sacubitril/valsartan, when assessed against the main inclusion/exclusion criteria for PARADIGM-HF. This wide range of eligibility depends on what background dose of ACEI/ARB must be attained before switching to sacubitril/valsartan. In this respect, some guidelines are more restrictive (e.g., those of the European Society of Cardiology which require patients to be up-titrated to an optimal dose of ACEI/ARB) whereas the regulatory labeling for sacubitril/valsartan in both the European Union and USA is more liberal (i.e., it does not require any specific dose of ACEI/ARB or even prior ACEI/ARB treatment at all) [6, 7]. Consequently, there is uncertainty about how to deal with the question of background ACEI/ARB dosing in studies of this type [8]. As demonstrated in multiple prior studies, many patients in the community are on lower than the “target” doses of ACEI or ARBs, recommended on the basis of randomized controlled trials [9–11]. However, as shown in PARADIGM-HF, and more recently in the Aliskiren Trial to Minimize OutcomeS in Patients with HEart failuRE trial (ATMOSPHERE) [12], many patients treated with a lower dose of ACEI/ARB can be up-titrated to a higher dose (and the findings in these trials are supported by prior trials adding an ARB to an ACEI) [13, 14]. In order to enter the run-in period in PARADIGM-HF, patients were required to have been treated with a stable dose of an ACEI or an ARB, equivalent to enalapril 10 mg/day, for at least 4 weeks before the screening visit [5]. In the run-in, 10,513 patients were treated with enalapril 10 mg bid for a median of 15 days. During that period, 10.5% of patients stopped enalapril, although discontinuation was due to an adverse effect or abnormal laboratory result in only 6.2% of patients [1]. In SwedeHF, around half of patients were not prescribed the target dose of 10 mg enalapril twice daily or equivalent and “ongoing up-titration” and “unknown” were the commonest reasons recorded for failure to reach target dose. Therefore, it was uncertain what denominator population we should use with respect to background ACE/ARB dose—patients on no or any dose, patients on at least enalapril 10 mg daily (or equivalent), or only those taking enalapril 20 mg daily or greater (or equivalent)? We decided to carry out all three analyses and found that the results were similar, irrespective of denominator population (although the size of the denominator population varied greatly). Specifically, 74% of patients taking any background ACEI/ARB dose were eligible, 75.5% of those taking ≥ 10 mg enalapril or equivalent daily, and 77.4% of participants taking ≥ 20 mg enalapril or equivalent daily, based upon analysis of patients with complete information. Of course, since only 78% of patients were taking ≥ 10 mg enalapril or equivalent daily and only 55% ≥ 20 mg enalapril or equivalent daily, only 58.9% of all patients were eligible for sacubitril/valsartan based upon an enalapril dose of ≥ 10 mg or equivalent daily (and 42.6% based on an enalapril dose of ≥ 20 mg or equivalent daily) using the non-imputed data (these proportions were 52% and 35%, respectively, using the imputed dataset). The analysis using the largest denominator (potentially eligible patients, irrespective of ACEI/ARB dose), giving an eligible proportion of the overall population of 74%, is consistent with application of the regulatory labeling whereas the analysis using the smallest denominator (only patients taking ≥ 20 mg enalapril or equivalent daily considered potentially eligible), giving an eligible proportion of the overall population of 34%, is more consistent with the ESC guidelines [2].

It is of interest that the overall proportion of patients ineligible for other reasons was quite similar, irrespective of background ACEI/ARB dose. The most common reason for ineligibility was a low natriuretic peptide level, with between 13.7 and 16.1% of patients excluded for this reason, depending on denominator population. However, a low natriuretic peptide concentration was, *relatively*, a more common exclusion in patients treated with a higher dose of ACEI/ARB, as might be expected [15]. This is an interesting finding given the disagreement between guidelines and the divergence between guidelines and regulatory labeling, with respect to whether sacubitril/valsartan use should be restricted to patients with an elevated natriuretic peptide level. A natriuretic peptide level threshold was used in PARADIGM-HF to ensure an adequate event rate in the trial and the benefit of sacubitril/valsartan over enalapril was consistent across the range of baseline NT-proBNP concentrations in PARADIGM-HF [16]. There is therefore no biological basis for restricting the use of sacubitril/valsartan to a particular natriuretic peptide level, although the absolute risk reduction with treatment is likely to be greater in patients with a higher NT-proBNP concentration as such patients are at higher absolute risk [16].

The next most frequent reason for ineligibility was low systolic blood pressure, and an opposite directional pattern to that seen for NT-proBNP was observed, i.e., this exclusion was *less* common in patients taking *larger* doses of ACEI/ARB (5.9% in the ≥ 20 mg dose group, 7.9% in the ≥ 10 mg dose group, and 9.1% in the any dose of ACEI/ARB group). When considering the absolute proportions of patients excluded because of low blood pressure, it should be noted that our analysis was conservative as we used a threshold of 100 mmHg which was the threshold for entry into screening in PARADIGM-HF whereas the threshold for randomization was 95 mmHg [5].

A similar pattern to that seen for blood pressure was observed for renal dysfunction—with 0.83% of the largest ACEI/ARB dose group ineligible for this reason compared with 3.6% of those taking any dose of ACEI/ARB. However, renal dysfunction, overall, was an infrequent contraindication to use of sacubitril/valsartan.

Hyperkalemia was an even less common contraindication to sacubitril/valsartan, with no clear gradient across the ACEI/ARB dose categories (1.2% in the highest dose category compared with 1.4% among patients taking any dose).

The observation in PARADIGM-HF that patients randomized to the ARNI had a lower risk of renal impairment and hyperkalemia during follow-up, compared with enalapril, is of interest when thinking about switching a patient with a lower eGFR or higher potassium level from an ACEI/ARB to sacubitril/valsartan [1].

By definition, the patients who were identified as potentially eligible for sacubitril/valsartan had higher natriuretic peptide levels, higher systolic blood pressure and eGFR, as well as a lower serum potassium concentration. Less intuitively, eligible patients were older and more often had a history of hypertension (both associated with a higher blood pressure and natriuretic peptides), atrial fibrillation, and valvular disease (both associated with higher natriuretic peptides). Less easy to explain is the finding that eligible patients were more likely to have heart failure of shorter duration than patients not eligible for sacubitril/valsartan.

There is one other report about eligibility for sacubitril/valsartan from a single center in the United Kingdom. In that study, Pellicori and colleagues identified 1396 patients with heart failure and a reduced EF with a contemporaneous measurement of NT-proBNP, of which only 379 (27%) were already on target dose of an ACEI/ARB [8]. The authors were not able to apply the NT-proBNP inclusion criteria as in the trial (i.e., ≥ 600 pg/ml or, in patients hospitalized for heart failure in the preceding 12 months, ≥ 400 pg/ml), because timing of hospitalization was not known. However, using a 400 pg/ml threshold, 66 of 379 patients (17.4%) would have been ineligible, similar to the 16.1% excluded for this reason in our equivalent population [8]. Low blood pressure and eGFR and high potassium were less common causes of ineligibility for treatment with sacubitril/valsartan, similar to what we found in SwedeHF.

There are also two reports about use of sacubitril/valsartan from the Get With the Guidelines-Heart Failure (GWTG-HF) initiative in the USA. One report examined eligibility for treatment in registry patients hospitalized with HF-REF between January 2011 and December 2013. Overall, 69% met FDA labeling criteria for sacubitril/valsartan and 55% of these individuals met PARADIGM-HF-like eligibility criteria (38% of patients overall) [17]. Although this was a study of hospitalized patients, the proportions eligible were similar to those identified in our analyses of ambulatory patients in a European country. The other study examined actual sacubitril/valsartan prescribing among 21,078 discharges from 347 hospitals [18]. This treatment was prescribed in only 495 cases (equivalent to 3.6% of discharges from January to June 2016). This much lower rate of actual use (as opposed to potential use) may reflect the difference between the ambulatory patients enrolled in PARADIGM-HF and the hospitalized patients in GWTG-HF, as well as barriers to prescribing [17].

### Limitations

We used only the most recent patient encounter recorded in SwedeHF. Eligibility/ineligibility for sacubitril/valsartan is not fixed and is likely to change over time reflecting the fluctuating nature of heart failure. Consideration of a patient for sacubitril/valsartan, as with any drug or device therapy, should be re-evaluated several times during the patient journey. For example, implantation of cardiac resynchronization therapy could improve blood pressure or renal function in a patient. The PARADIGM-HF EF inclusion criterion (≤ 40%) did not exactly match the EF categories recorded in SwedeHF (< 30%, 30–39%, 40–49%, and > 50%).

### Conclusions

Between 34 and 74% of patients with heart failure and reduced EF are eligible for treatment with sacubitril/valsartan, depending on the background dose of ACEI/ARB deemed necessary before switching therapy. The other main inclusion/exclusion criteria used in PARADIGM-HF render around 20 to 30% of patients ineligible for sacubitril/valsartan and this proportion does not differ much by background dose of ACEI/ARB.

## Electronic supplementary material

ESM 1 (DOCX 15 kb)
