# Supplementary material for: “Real World” Eligibility for Sacubitril/Valsartan in Unselected Heart Failure Patients: Data from the Swedish Heart Failure Registry
Source: Cardiovasc Drugs Ther. 2019 Mar 23;33(3):315–22. doi: 10.1007/s10557-019-06873-1 (PMC6538576; doi:10.1007/s10557-019-06873-1)
Supplement: Supplementary file 1 — (DOCX 15 kb) [file 10557_2019_6873_MOESM1_ESM.docx]

**Supplementary Appendix**

Sensitivity Analyses: All patients in Swedish Heart Failure Registry irrespective of setting of recruitment

Of 51,060 patients, 1875 were excluded because of death index admission or on day of visit, a further 19,043 because of an ejection fraction greater than 40% and among the remainder, 2045 were excluded because they were NYHA functional class I. This left 28,097 patients potentially eligible for treatment with sacubitril/valsartan, irrespective of setting of recruitment.

| *Table 1 Eligibility scenarios irrespective of setting of recruitment (inpatient or outpatient)* | | | |
| --- | --- | --- | --- |
|  | Prescribed any or no ACEI/ARB  (n= 28097) | Prescribed ≥ 10mg enalapril/d or equivalent  (n=17332) | Prescribed ≥ 20mg enalapril/d or equivalent  (n=10341) |
| Patients with complete data | 5376 (19.1) | 3971 (22.9) | 2647 (25.6) |
| ***Reason for ineligibility*** | | | |
| Natriuretic peptide criteria | 594 (11.0) | 505 (12.7) | 374 (14.1) |
| SBP <100 mmHg | 512 (9.5) | 320 (8.1) | 169 (6.4) |
| eGFR <30 ml/min/1.73m^2^ | 299 (5.6) | 112 (2.8) | 38 (1.4) |
| K^+^ >5.2 mmol/L | 82 (1.5) | 59 (1.5) | 35 (1.3) |
| Any of above | 1372 (25.5) | 927 (23.3) | 571 (21.6) |
| Values displayed as n (%)  ACE, angiotensin converting enzyme; ARB, angiotensin receptor blocker; GFR, glomerular filtration rate; SBP, systolic blood pressure | | | |

| *Table 2 Extent of missing data in SwedeHF* | | |
| --- | --- | --- |
|  | Missing (%) | Complete (%) |
|  |  |  |
| Age (years) | 0 (0%) | 12866 (100%) |
| Gender | 0 (0%) | 12866 (100%) |
| Duration of HF | 39 (0.3%) | 12827 (99.7%) |
| Systolic BP (mmHg) | 251 (2%) | 12615 (98%) |
| Diastolic BP (mmHg) | 277 (2.2%) | 12589 (97.8%) |
| Heart rate (bpm) | 814 (6.3%) | 12052 (93.7%) |
| BMI (kg/m^2^) | 7177 (55.8%) | 5689 (44.2%) |
| NYHA functional class | 1850 (14.4%) | 11016 (85.6%) |
|  |  |  |
| ***Medical History*** | | |
| Hypertension | 421(3.3%) | 12445 (96.7%) |
| Atrial fibrillation/flutter | 91 (0.7%) | 12775 (96.7%) |
| Ischaemic heart disease | 647 (5.0%) | 12219 (95.0%) |
| Valvular heart disease | 521 (4.0%) | 12345 (96.0%) |
| Left bundle branch block | 2204 (17.1%) | 10662 (82.9%) |
|  |  |  |
| ***Treatment*** | | |
| ACE inhibitor status | 26 (0.2%) | 12840 (99.8%) |
| ACE inhibitor agent | 4296 (33.4%) | 8570 (66.6%) |
| ACE inhibitor dose | 4296 (33.4%) | 8570 (66.6%) |
| Angiotensin receptor blocker status | 28 (0.2%) | 12838 (99.8%) |
| Angiotensin receptor blocker agent | 9288 (72.2%) | 3578 (27.8%) |
| Angiotensin receptor blocker dose | 9288 (72.2%) | 3578 (27.8%) |
| Beta-blocker status | 31 (0.2%) | 12835 (99.8%) |
| Diuretic status | 47 (0.4%) | 12819 (99.6%) |
| Digoxin status | 60 (0.5%) | 12806 (99.5%) |
| Mineralocorticoid receptor antagonist | 63 (0.5%) | 12803 (99.5%) |
| Device therapy (any of pacemaker, CRT-P, CRT-D, ICD) status | 151 (1.2%) | 12715 (98.8%) |
|  |  |  |
| ***Laboratory values*** | | |
| Haemoglobin (g/dL) | 0 (0%) | 12866 (100%) |
| Creatinine (µmol/L) | 33 (0.3%) | 12833 (99.7%) |
| Potassium (mmol/L) | 3629 (28.2%) | 9237 (71.8%) |
| NT-proBNP (pg/ml) | 7838 (60.9%) | 5028 (39.1%) |
| BNP (pg/ml) | 12007 (93.3%) | 859 (6.7%) |
| ACE, angiotensin converting enzyme; BP, blood pressure; BMI, body mass index; BNP, brain natriuretic peptide; CRT, cardiac resynchronization therapy; HF, heart failure; ICD, implantable cardioverter defibrillator; NYHA, New York Heart Association | | |
